# Clinical outcome and quality of life after reoperative CABG: off-pump versus on-pump – observational pilot study

**DOI:** 10.1186/1749-8090-8-66

**Published:** 2013-04-05

**Authors:** Engin Usta, Raoof Elkrinawi, Adrian Ursulescu, Ragi Nagib, Martin Mädge, Schahriar Salehi-Gilani, Ulrich FW Franke

**Affiliations:** 1 Department of Cardiac and Vascular Surgery, Robert-Bosch-Hospital, Auerbachstr 110, Stuttgart D-70376, Germany

## Abstract

**Background:**

Coronary artery bypass grafting (CABG) on cardiopulmonary bypass (CBP) is associated with significant morbidity and mortality. In high-risk patients, doomed for reoperation the adverse effects of CBP may be more striking. We evaluated the results of reoperative CABG (redo-CABG) by either off-pump (OPCAB) or on-pump (ONCAB). Clinical endpoints were perioperative myocardial infarction, mortality, survival and as the most striking difference between prior studies the quality of life (QoL).

**Methods:**

We performed a prospective, non-randomized assessment for patients who underwent redo-CABG by redo-OPCAB (n = 40) or redo-ONCAB (n = 40) at our institution between January 2007 and December 2010. For evaluation of QoL the SF-36 health survey was used with self-administered assessment.

**Results:**

During follow-up 37 of 40 patients were alive in the redo-OPCAB group versus 32 of 40 patients in the redo-ONCAB group (p < 0.05). The shorter operation time, less blood loss, fewer perioperative myocardial infarctions, the higher rate of totally arterial revascularisation and shorter intensive care stay were the significantly beneficial differences for patients in the redo-OPCAB group (p < 0.05). The 3-year survival rate was higher in the redo-OPCAB group with 81 ± 12% versus 63 ± 9%in the redo-ONCAB group. The quality of life survey did not reveal any significant differences between both groups.

**Conclusion:**

In conclusion, with our present retrospective study, we could demonstrate the safety and efficacy of the redo-OPCAB technique with even higher 3-year survival rate. Both techniques seem to have similar impact on the outcome of patients.

## Background

Coronary artery bypass grafting (CABG) of high-risk patients with the use of the cardiopulmonary bypass (CPB) may be associated with deleterious effects, including the diffuse systemic inflammatory response, global myocardial ischemia and the risks of aortic manipulation [1,2]. These may contribute to the morbidity associated with CABG [3]. Off-pump coronary artery bypass grafting (OPCAB) has emerged as an alternative technique to on-pump coronary artery bypass grafting (ONCAB) allowing coronary revascularization without the risk associated with CPB [4]. The OPCAB approach has been shown to reduce the overall systemic inflammatory response, including cytokine-mediated response [5,6]. It also avoids physiologic derangement to the heart caused by cardioplegic arrest as shown by reduced troponin T release [5,7].

As operative techniques evolve and survival after cardiac operations improves, the number of patients who have repeat sternotomy inevitably continues to increase. When coronary reoperation is necessary, the presence of a patent left internal mammary artery (LIMA) graft to the left anterior descending artery (LAD) creates specific risks, including the possibility of intraoperative injury to the graft and potential difficulties with myocardial protection [8,9]. Conventional redo-ONCAB after previous CABG represents a surgical challenge due to the potential for injury to patent coronary grafts, aorta or right ventricle and has been associated with increased mortality and morbidity [10-12]. The mortality rate after redo-ONCAB is reported to be more than three times higher than that of primary CABG [12]. Injury to a patent LIMA-LAD graft at reoperation can have catastrophic outcomes.

In this study, we retrospectively analyzed our results of redo CABG either as redo-OPCAB or redo-ONCAB in non-randomized patients. The endpoints evaluated for the study were: in-hospital mortality, early mortality, late mortality, follow-up survival, hospital stay, intensive care stay, rate of totally arterial revascularization, incidence of perioperative myocardial infarction, perioperative stroke, major adverse cardiac events, postoperative renal failure, postoperative complications and transfusion of blood or blood products. Additionally, we explored the endpoints actual health status and quality of life (QoL). The recurrence of cardiac disorders like dyspnea according NYHA III and angina pectoris according CCS III were also evaluated. The results should clarify if clinical outcomes differ depending on the chosen operation for this selected high-risk patient group.

## Methods

### Patient selection

The study protocol followed the Declaration of Helsinki and was approved by the Ethics and Research Committee of the Robert-Bosch-Hospital in Stuttgart Germany an academic training hospital of the Tuebingen University. Between January 2007 and December 2010 80 patients underwent reoperative CABG (redo-CABG) in the Departement for Cardiac- and Vascular Surgery of the Robert-Bosch-Hospital in Stuttgart Germany. A consecutive prospective series of n = 40 patients were operated off-pump (redo-OPCAB group) and n = 40 patients on-pump (redo-ONCAB group). A randomization which procedure is chosen for the patient was not applied. Data analysis indicate that in the beginning more patients were operated on-pump resulting in different follow-up times and that patients with renal insufficiency were operated preferentially off-pump (Table 1). After obtaining institutional review board approval, medical records, including operative notes, were retrospectively reviewed for all patients who underwent redo-CABG in the above mentioned period.

**Table 1:** Presents patients demographic data

**Variable**	**Redo-OPCAB (n = 40)**	**Redo-ONCAB (n = 40)**	**p-value**
**Age (years)**	72 ± 10	71 ± 9	0.12
**Gender**			
**Male**	32	36	0.07
**Female**	8	4	0.07
**Body mass index (kg/m2)**	28 ± 4	29 ± 4	0.91
**Left-ventricular ejection fraction (%)**	52 ± 12	53 ± 18	0.97
**Comorbidities**			
**Diabetes mellitus (%)**	43 ± 50	39 ± 49	0.59
**Hypertensive disease (%)**	100	96 ± 9	1.00
**Smoking history (%)**	65 ± 48	61 ± 5	0.73
**Previous CABG (months ago)**	140 ± 74	139 ± 69	0.80
**Previous coronary grafts (n=)**	3 ± 1	3 ± 1	0.05
**Euroscore (logistic)**	23 ± 19	22 ± 17	0.66
**Creatinine (mg/dl)**	1.2 ± 1.2	1.0 ± 0.21	0.65

OPCAB, off-pump coronary artery bypass grafting; ONCAB, on-pump coronary artery bypass grafting; NYHA class, New York Heart Association classification; CCS class, Canadian Cardiovascular Society classification; Data are expressed as mean ± standard deviation.

We sent each patient enrolled into this study a self-administered questionnaire about the actual health situation. Additionally, each patient received a self-administered Short Form Health Survey (SF36) [13] giving information about the patient´s physical and mental status of the last four weeks. Nevertheless, inclusion criteria were living patients and those who completed the questionnaire and SF36 survey.

### Clinical endpoints

Patients of the redo-OPCAB and redo-ONCAB group were analysed by comparing various preoperative, perioperative and postoperative variables (Tables 1, 2, 3 and 4). Endpoints for the study were: in-hospital mortality, late mortality, blood loss, transfusion of blood or blood products, rate of totally arterial revascularization, perioperative myocardial infarction, length of intensive care stay, length of hospital stay, cumulative survival and composite event-free survival (Table 1).

**Table 2:** Procedural details, the postoperative course and information about the occurrence of perioperative myocardial infarction are depicted

**Variable**	**Redo-OPCAB (n = 40)**	**Redo-ONCAB (n = 40)**	**p-value**
**Operation time (min)**	**195 ± 69**	**248 ± 40**	**0.01**
**Total arterial revascularization (n=)**	**28**	**12**	**0.01**
Bilateral internal mammary artery (BIMA) (n=)	0.28 ± 0.45	0.30 ± 0.47	0.484
Radial artery	0.15 ± 0.43	0.19 ± 0.40	0.821
**Saphenic vein graft (n=)**	**0.3 ± 0.48**	**0.85 ± 0.72**	**0.007**
Single grafts (n=)	1.05 ± 0.55	1.52 ± 1.16	0.06
Sequential grafts (n=)	0.52 ± 0.55	0.62 ± 0.86	0.952
**Total blood loss (ml)**	**540 ± 494**	**867 ± 619**	**0.02**
**Transfusion of blood and/or derivatives (n=)**	11	9	0.53
**Postoperative ventilation (hours)**	7 ± 8	9 ± 10	0.08
**Intensive care stay (days)**	**3 ± 2**	**5 ± 3**	**0.01**
**In-hospital stay (days)**	15 ± 10	13 ± 5	0.88
**Cardiac ischemia markers (12 hours postop)**			
**CK (U/l)**	**335 ± 341**	**709 ± 746**	**0.01**
**CK-MB (U/l)**	38 ± 45	30 ± 37	0.78
**Troponin**	**0.5 ± 0.5**	**5.3 ± 11**	**0.01**
**LDH**	**208 ± 89**	**323 ± 135**	**0.01**
**Postoperative major cardiac adverse effects (n=)**	3	4	0.78
**Impaired wound healing (%)**	7 ± 3	4 ± 2	0.15

OPCAB, off-pump coronary artery bypass grafting; ONCAB, on-pump coronary artery bypass grafting; CK, creatine kinase; CK-MB, creatine kinase isoenzyme form MB; LDH, lactate dehydrogenase. Data are expressed as mean ± standard deviation. Significant values are printed in bold type.

**Table 3:** The actual follow-up and various forms of mortality are presented

**Variable**	**Redo-OPCAB (n = 40)**	**Redo-ONCAB(n = 40)**	**p-value**
**Actual follow-up (months)**	22 ± 11	27 ± 15	0.07
**Alive (n=)**	**37**	**32**	**0.04**
**In hospital mortality (n=)**	0	3	0.15
**Early mortality (n=)**	0	0	1
**Late mortality (n=)**	3	5	0.35
**Dyspnea according NYHA III (n=)**	5	4	0.14
**Angina pectoris according CCS III (n=)**	4	3	0.14
**Recent coronary reintervention (n=)**	**0**	**5**	**0.04**
**Composite events**	**8**	**17**	**0.02**

OPCAB, off-pump coronary artery bypass grafting; ONCAB, on-pump coronary artery bypass grafting. Data are expressed as mean ± standard deviation. Significant values are bold typed.

**Table 4:** Features the quality of life or SF-36 health survey in the redo-OPCAB and redo-ONCAB patients

**Variable**	**Redo-OPCAB (n = 40)**	**Redo-ONCAB (n = 40)**	**p-value**
**Physical performance**	58 ± 30	55 ± 30	0.65
**Role performance**	48 ± 43	49 ± 45	0.97
**Bodily pain**	65 ± 35	53 ± 30	0.15
**General health**	56 ± 23	51 ± 24	0.58
**Vitality**	48 ± 25	47 ± 26	0.98
**Social functioning**	74 ± 26	69 ± 31	0.67
**Role emotionale**	70 ± 39	76 ± 36	0.66
**Mental health**	77 ± 20	68 ± 18	0.06

OPCAB, off-pump coronary artery bypass grafting; ONCAB, on-pump coronary artery bypass grafting; Data are presented as mean ± standard deviation. Significant values are depicted in bold type.

In all enrolled patients the operations were performed by five staff surgeons, who are involved in our minimal invasive surgery program. These surgeons performed similar numbers of CABG but with different experience and learning curves regarding redo-OPCAB.

### Anesthesiological and haemodynamic management

The procedures were performed under general endotracheal anaesthesia with a regular single-lumen intubation. The patients were positioned on a bair hugger® mattress (Arizant Healthcare Inc., Eden Prairie, MN 55344, USA) in the operation room.

Hemodynamic monitoring comprised of six-channel electrocardiography with ST-segment trend analysis and radial arterial pressure. Monitoring included regular continuous pulse oxymetry.

In the redo-OPCAB group the FloTrac sensor and Vigileo^TM^ monitor (Edwards Lifesciences, Irvine, CA, USA) were utilized for minimal-invasively hemodynamic monitoring. This system calculates cardiac output by analyzing arterial pressure waveform [14,15]. In the Redo-ONCAB group we used pulmonary artery catheters to measure cardiac output by thermodilution technique. Recorded variables were mean arterial pressure, central venous pressure, mean pulmonary pressure, pulmonary capillary wedge pressure, cardiac index, cardiac output, and systemic vascular resistance. Arterial blood gases and activated clotting time (ACT) were monitored every 30 minutes.

During the operation a transesophageal echocardiography (TEE) (CX50 CompactXtreme, Philips, Germany) was utilized for assessment of myocardial wall irregularities and unclear related walve pathologies especially in the Redo-ONCAB group if weaning of CPB was protracted.

The anticoagulation regimen in the Redo-OPCAB group and Redo-ONCAB group differed. Intravenous heparin was applicated with a dose of 150 IU/kg body weight in the redo-OPCAB group, less than in the redo-ONCAB group with a dose of 300 IU/kg body prior to the institution of CPB to accomplish an activated coagulation time of greater than 400 sec. This different anticoagulation regimen, with lower heparin dose in the off-pump group derives from our clinical routine and is in accordance to data from literature [16] as cannulation of the aorta and right atrium is not necessary in the off-pump technique. Finally, the total amount of heparin was reversed with protamine after completion of the anastomoses.

### Surgical technique for redo-OPCAB

After median sternotomy the left internal mammary artery (LIMA) and or the right internal mammary artery (RIMA) were harvested by standard technique using hemoclips in a scletonizing fashion if not being utilized in the prior operation. In case of open graft vessels those were mobilized in their proximal segments. The other conduits (saphenous veins and radial artery) were harvested simultaneously using endoscopic vessel harvesting (EVH) (Maquet Inc., Rastatt, Germany). We used CTS OPCAB access platform and stabilizer (Cardiothoracic Systems, Cupertino, CA, USA) or Hercules Universal Stabilizer Arm™ with stabilizer (Estech Company, San Ramon, CA, USA) to stabilize the target coronary vessel. In the Redo-OPCAB group we always used intracoronary shunts (Baxter AnastaFLO intravascular shunt, Irvine, CA, USA) for anastomosis. In most cases, the left anterior descending coronary artery was the first coronary artery to be grafted. The vessels on the lateral and posterior wall were grafted later. However, the sequence of grafting was individualized for a particular patient, depending on the severity of the lesions in different coronary arteries and patient’s hemodynamics. Both the left anterior descending and right coronary arteries could be grafted without much displacement of the heart. For exposure of the circumflex and right coronary vessels pericardial traction sutures were used to pull the heart vertically. In most cases the pleurae were not opened. A vertical pericardiotomy was performed to herniate the heart to the right chest under the sternum. Other maneuvers such as the Trendelenburg position and tilting the table were performed as required. Inotropic agents were used when necessary during surgery. We used 8–0 Seralon polyamide suture (Serag-Wiessner KG, Naila, Germany) for the distal anastomosis and 6–0 for the proximal anastomosis.

### Surgical technique for redo-ONCAB

Conventional coronary artery bypass procedures were performed using standard CPB, which was established using ascending aortic and two-stage venous cannulation. An open extracorporeal circuit was used, which was identical in each patient and consisted of a membrane oxygenator, arterial filter and crystalloid priming. The LIMA and RIMA were harvested in a scelotonizing fashion if not being utilized in the prior operation. Open LIMA or RIMA in-situ graft vessels were mobilized in their proximal segments for the later cross-clamping during cardioplegia. The radial artery and saphenous vein were usually harvested utilizing endoscopic vessel harvesting equipment (EVH) (Maquet Inc., Rastatt, Germany). After instituting CPB the aorta and open LIMA or RIMA in-situ grafts were cross-clamped and the procedure was performed on an cardioplegic arrested heart. The patient was not actively cooled but temperature was allowed to drift as was usually between 32-34°C. Antegrade Brettschneider´s crystalloid cardioplegia (1500 ml) was used for myocardial protection (Dr. Franz Köhler Chemie Inc., Bensheim, Germany). Cardioplegia was repeated if myocardial contractions occurred. Rewarming was initiated during completion of the last peripheral anastomosis. Reperfusion was performed with whole blood by declamping of the aorta and open LIMA or RIMA in-situ grafts. Reperfusion time was defined till the end of CPB.

### Graft vessel selection

Routinely, all patients underwent preoperative clinical and non-invasive angiological examinations of the radial artery diameters (luminal diameter) by the attending cardiovascular surgeon. Further a normal Allen’s test on the chosen side was compulsory. Radial arteries with immense atherosclerotic changes or luminal diameter less than 2 mm were excluded prior to harvesting. The saphenous vein was scanned additionally and was doomed as a graft if the luminal diameter was at least 3 mm. Duplex ultrasound was performed by the cardiovascular surgeon with a Siemens ACUSON Antares™ ultrasound system equipped with a VF13-5 transducer probe (Siemens AG, Erlangen, Germany). A 2-D linear electronic probe at 7.0 MHz, pulse wave Doppler at 5.0 MHz and color Doppler at 5.0 MHz were used. The angle of the emitted Doppler ultrasound wave from the probe was adjusted to 60° to achieve the Doppler signal of the strongest intensity. The internal diameter of the vessel was measured using M-mode technique. Whenever feasible we favourized harvesting arterial grafts if not utilized in the past. Due to the prospective non-randomized character of this study the majority of the redo-ONCAB patients were operated in the first half of the observation period resulting in lesser utilization of arterial grafts in comparison to the redo-OPCAB patients. Further each patient was examined by coronary angiograpy assassing the viability of the native coronary arteries and the graft vessels preoperatively.

### Quality control and assessment criteria

Graft patency and bypass fow rates were analyzed intraoperatively using a handheld 2, 3 or 4 mm transit-time flowprobe connected to a MediStim VeriQ System (MediStim ASA, Oslo, Norway). Postoperative ECG (electrocardiogram), serial samples of creatine kinase (CK), CK-MB, cardiac troponin I (cTnI) and after 2009 high-sensitive cardiac troponin I (hs cTnI) were determined every 6 h up to 48 h in any patient. Myocardial infarction was defined as either Q-wave in the postoperative ECG or an increase in the CK-MB enzyme fraction above 10% and simultaneous increase of cTnI of more than 10 ng/ml or hs cTnI of more than 100 ng/ml. An early postoperative control angiography was not recommended, except for patients with postoperative myocardial infarction suspected (patients with postoperative angina and increase of troponin I and CK-MB levels).

### Postoperative patient management

Patients of both groups were transferred to the intensive care unit (ICU) and ventilation was kept till reaching a stabile haemodynamic situation. Usually, patients were weaned from ventilation as early as possible. On the next day, we transferred the patients to our intermediate care unit (IMC) for further surveying. We transferred the patients on postoperative day (POD) two our regular cardiovascular service till discharge from hospital.

### Postoperative follow-up and SF-36 health survey

We aimed to assess the surveillance and quality of life of the operated patients by sending them self-administered questionnaires by mail. Usually, we sent them a cover letter, the SF-36 questionnaire, a self-administered questionnaire on angina pectoris and dyspnea and a stamped self-addressed return envelope. We instructed our patients to give information about their health situation of the last four weeks.

The questionnaires consisted of two parts. Part one comprised items about the actual health situation and the recurrence of cardiac disorders like angina pectoris and dyspnea with necessary interventions in the follow-up period. The assessment of angina pectoris and dyspnea by self-administered questionnaire was considered valid in view of the very satisfactory agreement between the coding of the patient and medical coding (New York Heart Association (NYHA) and Canadian Cardiovascular Society classification (CCS) [17] for angina pectoris and for dyspnea [18].

Part two, the SF-36 health survey is one of the most extensive standardized, self-administered, generic questionnaires for measuring both the physical and mental health of a patient [5,13,19]. It was developed to assess the functional status and well-being of patients. SF-36 consists of 36 questions in eight areas: physical functioning (physical limitations in performance of daily living), role-physical (problems encountered with daily activities or work as a result of physical health), bodily pain (overall pain severity), general health perception (overall general health), vitality (frequency of feeling full of energy vs. tired), social functioning (performing normal social activities or not), role-emotional (problems with work or daily activities as a result of emotional problems) and mental health (degree of nervousness or depression). Higher values on the transformed 0–100 scale for each health domain indicate better health status.

### Survival analyses

Patients medical-records were surveyed respectively the incidence of parameters like mortality peri- or postoperatively. The mortality in this period was defined as in-hospital mortality. Death occurring until 30 days postoperatively was defined as early and after this period as late mortality.

Like in the preceding section mentioned the questionnaires consisted of two parts. Part one comprised items about the actual health situation and the recurrence of cardiac disorders like dyspnea according NYHA III and angina pectoris according CCS III with necessary coronary interventions in the follow-up period. If the questionnaires were not sent back after one month patients were telephoned or the family physician was contacted. In addition, patient medical-records were studied if during the last hospital stay MACCE (Major Adverse Cardiac and Cerebrovascular Events) occurred. Dyspnea according NYHA III, angina pectoris according CCS III, MACCE, in-hospital mortality, early and late mortality occurring during the hospital-stay and follow-up were defined as a censored event.

Later Kaplan-Meier analysis and Cox proportional hazards models were used to calculate hazard ratios (HR) and 95% confidence intervals (CI), with adjustments for baseline patient and disease characteristics.

Survival analyses consisted of two parts. Part one was cumulative survival with the subgroup 3-year survival. The censored event was defined as death occurring as in-hospital mortality, early and late mortality during follow-up. Part two was the assessment of composite event-free survival with subgroup 3-year survival. The censored event was defined as dyspnea according NYHA III, angina pectoris according CCS III, MACCE, recent coronary intervention, in-hospital mortality, early and late mortality occurring during the hospital-stay and follow-up.

### Statistical analysis

Values were expressed as mean ± standard deviation. Continuous data were analyzed using the Wilcoxon´s rank test. The Kaplan–Meier method was used to analyse cumulative survival, 3-year survival and freedom from events (death, myocardial infarction and repeat intervention, angina, dyspnea and MACCE). The log rank test was used to compare the survival and freedom from events between redo-OPCAB and redo-ONCAB. Probability values (P) of less than 0.05 were considered significant. Generally we presented the exact P-value. Statistical analysis was performed using the JMP program (version 9, SAS Institute, Cary, North Carolina, USA).

## Results

### Evaluation of patients characteristics

The patients preoperative demographic data did not differ significantly (p < 0.05) between the redo-OPCAB and redo-ONCAB groups (Table 1). Both groups revealed similar preoperative cardiac function and similar prevalence of comorbidities.

### Procedural details and intraoperative data

There were significant (p < 0.05) differences in the redo-OPCAB und redo-ONCAB groups. The shorter operation time, more use of arterial grafts, and less blood loss were the significant differences in the redo-OPCAB group (Table 2). No significant difference existed for the transfusion of blood or derivatives between both groups (Table 2).

### Postoperative results and clinical outcome

Patients in the redo-OPCAB group differed significantly (p < 0.05) with shorter intensive care stay, lower CK, Troponin and LDH expression. No significant difference existed for the incidence of postoperative major cardiac adverse effects, impaired wound healing and in-hospital mortality (Table 2).

### Analysis of intraoperative patients characteristics

The groups significantly differed in the operation time (redo-OPCAB, 195 ± 69, and redo-ONCAB, 248 ± 40 min; p = 0.01) (Table 2). Another significant difference was the rate of totally arterial revascularization redo-OPCAB, 28, and redo-ONCAB, 12 patients; p = 0.02) (Table 2). Although not significant, slightly more patients undergoing redo-ONCAB had more single coronary bypass grafts (redo-OPCAB, 1.05 ± 0.55, and redo-ONCAB, 1.52 ± 1.16; p = 0.06.

### Comparison of postoperative data

The groups significantly differed in the amount of postoperative total blood loss (redo-OPCAB, 540 ± 494, and redo-ONCAB, 867 ± 619 ml; p = 0.02) (Table 2). There was no significance in the category transfusion of blood and/or derivatives (redo-OPCAB, 11 patients, and redo-ONCAB, 9 patients; p = 0.53) (Table 2).

Further significant differences were the intensive care stay (redo-OPCAB, 1.92 ± 1.71, and redo-ONCAB, 2.93 ± 2.37; p = 0.01). Although not significant, slightly more patients undergoing redo-ONCAB were ventilated longer (redo-OPCAB, 7.3 ± 8.2, and redo-ONCAB, 8.6 ± 5.5 hours; p = 0.08) (Table 2).

### Cardiac ischemia and MACCE

Considering the perioperative myocardial infarction parameters, there are significant differences in both groups with higher rate of infarction in the redo-ONCAB group (Table 3). Creatine kinase (CK) and troponin I levels were higher in the redo-ONCAB group (redo-OPCAB, 334.5 ± 341.4, and redo-ONCAB, 707.4 ± 745.7 U/l; p = 0.01) and (redo-OPCAB, 0.52 ± 0.48, and redo-ONCAB, 5.26 ± 11.18 ng/ml; p = 0.01), respectively (Table 3).

The only significant difference in the category of MACCE was the occurrence of postoperative brain organic disease, with significantly higher frequency in the redo-ONCAB group (redo-OPCAB, 0%, and redo-ONCAB, 15 ± 4% of patients; p = 0.02).

### Cumulative survival

**Figure 1:**
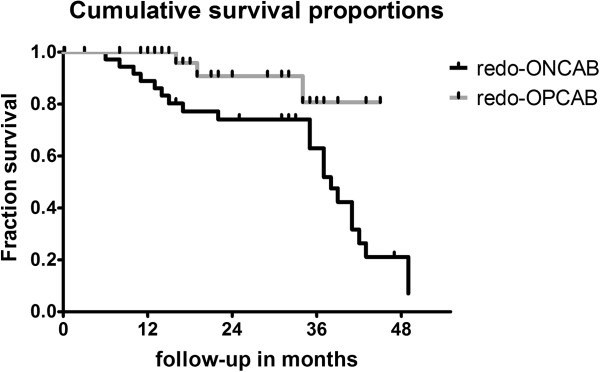
**Kaplan-Meier analyses of cumulative survival.** OPCAB, off-pump coronary artery bypass grafting; ONCAB, on-pump coronary artery bypass grafting. On the X-axis the follow-up in months and on the Y-axis the fraction survival are given. The censored event is death occurring during follow-up.

The Kaplan–Meier analyses are depicted in Figure 1. Although not significant, 3-year survival was 81 ± 11% in the redo-OPCAB and 63 ± 9% in the redo-ONCAB group (p = 0.08). In the log rank test with a Chi square of 5.88 the cumulative survival curves were significantly different (p = 0.01).

The hazard ratio was 2.99 with a 95% CI (confidence interval) of ratio 0.78–5.42.

### Composite event-free survival

**Figure 2:**
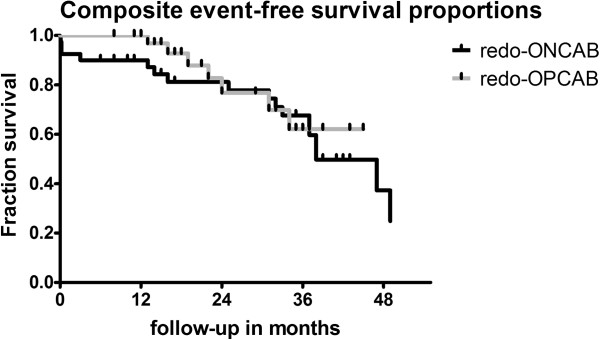
**Kaplan-Meier analyses of composite event-free survival.** OPCAB, off-pump coronary artery bypass grafting; ONCAB, on-pump coronary artery bypass grafting. On the X-axis the follow-up in months and on the Y-axis the fraction survival are given. The censored event was defined as death occurring as in-hospital mortality, early and late mortality during follow-up. The censored event was defined as dyspnea according NYHA III, angina pectoris according CCS III, MACCE, recent coronary intervention, in-hospital mortality, early and late mortality occurring during the hospital-stay and follow-up.

The Kaplan–Meier analyses are presented in Figure 2. Although not significant, 3-year composite event free survival was 62 ± 8% in the redo-OPCAB and 68 ± 8% in the redo-ONCAB group (p = 0.87). In the log rank test with a Chi square of 0.36 the cumulative survival curves were not significantly different (p = 0.55).

The hazard ratio was 1.32 with a 95% CI of ratio 0.54–3.21.

### Quality of life assessment and SF-36 health survey evaluation

**Figure 3:**
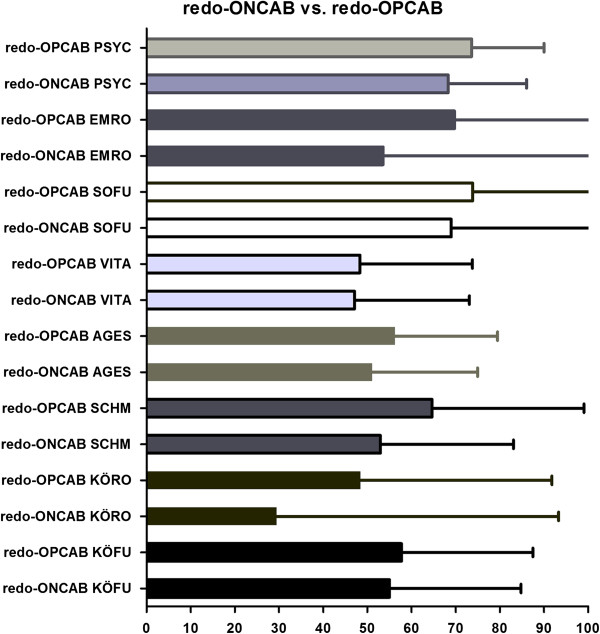
**Figure 1 presents the evaluation of the quality of life or SF-36 health survey in the redo-OPCAB and redo-ONCAB patients.** For this survey eight major categories were evaluated and the transformed scores were depicted on a 100 point score scale. PP, physical performance; RP, role performance; BP, bodily pain; GH, general health; VT, vitality; SF, social functioning; RE, role emotionale; MH, mental health; OPCAB, off-pump coronary artery bypass grafting; CABG, coronary artery bypass grafting; Data are expressed as mean ± standard deviation.

The redo-OPCAB and redo-ONCAB groups were evaluated on the basis of eight categories according to the SF-36 health survey (Figure 3). The categories were physical performance (PP), role performance (RP), bodily pain (BP), general health (GH), vitality (VT), social functioning (SF), role emotionale (RE) and mental health (MH). There were no significant differences among both groups. Although not significant, slightly more patients undergoing redo-OPCAB had higher scores in mental health (redo-OPCAB, 76.82 ± 19.91, and redo-ONCAB, 68.31 ± 17.34; p = 0.06) (Figure 3).

## Discussion

In an attempt to avoid the deleterious effects of cardiopulmonary bypass (CPB), off-pump coronary artery bypass grafting (OPCAB) has gained broader acceptance since the last decade. Nevertheless conventional non-beating heart on-pump CABG is still the standard technique used in coronary artery surgery in many institutions. Complications in relation to this technique are due to the release of inflammatory mediators, the use of cardioplegia, aortic cross clamping and hypothermia [20]. Blood contact with the artificial surfaces of the CPB circuit produces a well-documented diffuse inflammatory response that affects multiple organ systems. Virtually all detrimental effects of this diffuse inflammatory response increase with large duration of CPB.

OPCAB technique was introduced to avoid such complications. Despite its efficiency and safety over conventional CPB, the technique was criticized by many investigators regarding completeness of myocardial revascularization, graft patency and long term results. Coronary artery bypass grafting on the beating heart is a more demanding procedure than conventional CABG. It is more difficult in terms of technical skills and requires more attention to the surgeon and the anaesthesiologist. Many authors [3,21,22] have reported series of OPCAB. Although they have presented excellent mortality rates, concern has been raised over a decrement in graft patency rates [23]. One of the important draw backs of this technique is the hemodynamic deterioration which can occur during manipulation of the heart during surgery, which entails urgent transfer to conventional CPB [24,25].

Considering the above mentioned data, we have meanwhile established the OPCAB technique for coronary revascularization as our standard technique. Nowadays we perform at least more than 90% of primary coronary revascularizations by OPCAB (not yet published data). In the cases of redo coronary revascularisations we achieved similar rates utilizing OPCAB (not yet published data). Therefore our presented data contains a significantly difference in regard of the postoperative follow-up period in the redo-OPCAB and redo-ONCAB group. The explanation for this circumstance is that in the second half of the observation period most of the redo coronary revascularisations were performed by redo-OPCAB. As in the first half of the observation period more single saphenic vein grafts were anastomosed in the second half the trend was towards total arterial revascularization with sequential anastomosis. This may be the result of the maturation process or completing the learning curve and changes of staff surgeons with different experience and learning curves regarding redo-OPCAB. Despite of the referred drawbacks of the OPCAB technique [25], we had never to convert redo-OPCAB to redo-ONCAB in our study population. Interestingly, the number of previous coronary grafts determined preoperatively was higher in our redo-ONCAB group. Therefore the higher number of distal anastomosis just reflects the higher occlusion rate of previous coronary grafts in the redo-ONCAB group. Nevertheless, we have considered complete revascularization to be the “gold standard” for coronary bypass operations irrespective of the surgeon’s choice of incision or use of CPB, and still hold this belief.

The present study reports clinical outcomes and quality of life surveillance in 69 patients undergoing redo coronary revascularization either by redo-OPCAB or redo-ONCAB. After prior review of the appropriate literature we could not find any study investigating the quality of life of these highly selective patients group in such an extent. Our study aims to clarify if both procedures differ at the endpoints mortality, transfusion requirements, perioperative myocardial infarction, inotropic drug support, major adverse effects, hospital stay, quality of life and recurrence of cardiac disorders. Considering our study population with a mean Euroscore (logistic) around 23, it is justified to declare them as high risk patients. To our knowledge, it is unique and different from published data to include particularly the high risk patients in such a study [26,27]. In the present retrospective comparison, we demonstrate the safety and efficacy of redo-OPCAB. Redo-OPCAB patients, although generally having similar preoperative risk factors like on-pump redo-ONCAB patients, enjoy a decreased incidence of perioperative myocardial infarction, postoperative organic brain disease, and decisively a decreased mortality. Considering our data, we can claim that redo-OPCAB patients have shorter operation times, less postoperative blood loss and shorter stay on IMCU. Although not significant, postoperative ventilation tends to be shorter in the redo-OPCAB group. Our presented data is in analogy to those of the referred literature [26-31].

In contrast to the above presented data, the evaluation of the SF-36 health survey [13] did not reveal significant differences among the redo-OPCAB and redo-ONCAB groups.

### Limitations of the study

We want to address few limitations of our present study. The study comprises, in comparison to the referred data high-risk patients undergoing coronary redo revascularisation. Both groups differ in the follow-up period. The limitations of our study include the prospective single-institution methodology being not a randomized clinical trial. Further limitations are the small number of patients, the inability to conduct a reliable multivariate analysis of predictor variables of late mortality due to the small number of patients with the index event.

## Conclusions

In conclusion, with our present retrospective study, we could confirm the safety and efficacy of both surgical procedures of redo-CABG, with better results for the redo-OPCAB technique. Nevertheless in this era where the use of multivessel coronary angioplasty is extensively employed the therapeutic option of redo-CABG, tendencially declining, should not be ignored in our clinical routine. Our future effort will be to perform a double-blinded randomized prospective study to gain more information and to clarify which technique is superior in this selective patient group especially in regard to the quality of life.

## Patients’ consent

Written informed consent was obtained from the patient for publication of this report and any accompanying images.

## Competing interests

The authors declare that they have no competing interests.

## Authors’ contributions

UE carried out the patient evaluation and participated in the study design and coordination. UE performed the statistical analysis and created the manuscript. UE and ER were engaged in the data evaluation process. UA, NR, MM, S-G S and FUF carried out the redo coronary revascularisations by either method. FUF conceived of the study, and participated in its design and coordination. All authors read and approved the final manuscript.
